# Electricity and Life

**Published:** 1875-07

**Authors:** E. H. Gibbs


### ELECTRICITY AND LIFE.

BY E. H. GIBBS, M.D.

It was formerly supposed that there is a "nervous fluid" circulating through the entire system of nerves, as the blood flows through the arteries and veins. This fluid passes through the nerves with inconceivable velocity—more rapid than a ray of light in its course from the sun, even "as quick as lightning." This theory of the nervous fluid was long ago abandoned; and now the doctors talk of a nervous *force.* What this force really is, we are not yet told. It seems as if the wise men have simply adopted a change of terms, and that it is immaterial whether they talk of a nervous force or a nervous fluid. We get as clear and definite idea of the principle of life under the one name as by the other. Call it *force* or *fluid*, we can know it and study it only by the phenomena which it produces. And herein it is very like electricity. That mysterious agent is known by its effects only. It has neither weight, nor form, nor color, nor any of the qualities which our physical senses recognize. True, we see the spark and hear the crackling as it is discharged from the Leyden jar; we see the flash from the summer cloud and hear the thunder, and we say these are electrical phenomena, and that is all we know about it. We may talk learnedly about the positive and negative conditions of the jar and the cloud; we may tell the names of conductors and non-conductors; we may draw it silently from the sky, or we may evolve it by chemical action and send it around the world with our letters, but what do we know of *itself?* We boast that this, the most wonderful force in nature, has become our most obedient servant; but can we describe that servant in his personal appearance?

In short, we cannot tell what *it is;* but science has made wonderful progress in the last half century in finding out what it does.

It is not now our purpose to consider its *physical* effects; but we wish to refer briefly to a few of its operations on the living body. Some have thought this subtle agent is the principle of life; that the old idea of a nervous fluid is not an irrational one, and that that fluid is electricity. It is as easy to accept the later expression of nervous force, and then we shall be nearer the line of modern teaching if we claim that that force is electricity.

Perhaps the first of the vital phenomena of this agent was observed by an Italian philosopher, who found that the electric current passed through the leg of a frog produces muscular contractions. This experiment, familiar to the merest tyro in electrical science, has been repeated a thousand times. Then, in the course of time, came the practical studies of the effect of electricity on the human body. We remember the stories of executed criminals restored to a semblance of life by the galvanic battery. These, and many other similar experiments, demonstrated the remarkable physiological effect of this mysterious agent, and lead to the belief that here, indeed, was found the principle of life.

The medical use of electricity is of comparatively recent date. Years ago there was occasionally a physician who owned an electric machine; and sometimes when he had exhausted all his resources in the drug line in the attempt to cure or relieve some obstinate chronic disease he would resort to electricity. At least he would give his patient *shocks.* He treated his cases all in the same way, and that was simply to let the patient take a wire in each hand, and then the doctor turned the crank. That was all he could do, for he had no knowledge of the proper method of applying electricity in different diseases. His treatment was simply empirical. But now, such has been the progress in the study of the physiological and remedial effects of this agent, that we may confidently affirm that no branch of the physician's art rests on a more scientific basis than does that of medical electricity. Many of our well-educated and experienced practitioners have devoted themselves to its special study and practice. They have often succeeded in curing diseases that could not be reached by any other medication. They have learned that it is to the class of nervous affections that electricity is the most applicable. In these cases its curative results often approach the marvelous. And these form a large proportion of the diseases which the general practitioner is daily called upon to treat. As a people we are peculiarly liable to nervous disorders. Where is the man who has not at some time experienced a twinge of *neuralgia?* Find such an one who has always remained in blissful ignorance of what neuralgia is, and we will produce a score who can describe the symptoms of that disease, each in his own case, better than they are given in the books. This in some of its various forms, produces nine-tenths of the physical suffering of women.

And then we have the host of troubles that come from *nervous exhaustion.* The worn out man, the business man, whose constant cares and unceasing mental toil have drawn so hard upon his stock of vitality that his food gives him no nourishment, his sleep affords no rest and his mind is as unbalanced as his body, he knows what is meant by *nervous exhaustion.*

In these cases electricity is the restorative. It seems to be what iron is to one whose blood is thin and watery. It is perhaps as near a specific in such cases as is quinine in intermitent affections.

All the functions of life are dependent upon a healthy supply of the nervous influence.

Nervous derangement may cause indigestion, constipation, billious disorders, palpitation of the heart and difficult respiration, cough and croup and in short there is hardly a disease that may not be caused or simulated by some derangement of the nervous system. And this explains why electricity should cure so many different diseases. It gives to the nerves strength and vigor. It restores that essential *something*, without which they are like the veins and arteries after great losses of blood.

Rheumatism in its chronic form is often greatly relieved if not perfectly cured by the proper application of electricity. We would advise those who have suffered from this painful complaint and have found no permanent relief in medicines to avail themselves of the benefit which electricity is quite sure to afford.

The electric current when passed through the region of the liver and stomach and bowells act upon these organs as a gentle and natural stimulant.

That torpid condition of the liver, which results in the various ailments and disorders known under the comprehensive term of billious, is readily corrected by one or two applications of electricity. The most obstinate cases of constipation are often relieved by the same means.

But we cannot mention all the diseases for which this new and powerful remedy is indicated.

The more its effects are understood the more extensive becomes its range as a curative agent.

Some of the ablest scientific men in the world are, to-day, engaged in experimental studies of the physical, physiological and remedial powers of electricity. They are daily looking further and deeper into its mysteries; but the more they know of it the more they find there is yet to be learned. They are not now prepared to declare that this is the *principle of life*, but they believe that it is nearer to it than any other force, or substance or agency, material or immaterial, yet recognized in the domain of human knowledge.

---

Be temperate in all things.

